# Study on Road Performance and Substitution Rate Optimization of Highway Solid Waste Recycled Aggregate

**DOI:** 10.3390/ma19173757

**Published:** 2026-09-03

**Authors:** Zhenwei Lin, Hao Long, Yuanqing Liu, Qiu Zhao

**Affiliations:** 1 College of Intelligent Construction, Fuzhou Institute of Technology, Fuzhou 350506, China; lzw11559@gmiot.com; 2 College of Civil Engineering, Fuzhou University, Fuzhou 350108, China; 210527218@fzu.edu.cn (H.L.); 230520093@fzu.edu.cn (Y.L.)

**Keywords:** construction and demolition waste, substitution rate, highway reconstruction and expansion, roadbed fill, cement-stabilized recycled aggregate

## Abstract

In view of the differences in the specified substitution rates of construction and demolition waste (C&DW) recycled materials in road applications between China and other countries, this study focuses on a highway reconstruction and expansion project in Guangdong Province, China, and systematically analyzes the influence of the recycled aggregate substitution rate on the road performance of subgrade filler and cement-stabilized recycled aggregate (CSRA). Based on the current domestic JTG series specifications, combined with a literature review and comparative analysis, the effects of particle size, maximum dry density (MDD), optimum moisture content (OMC), and California bearing ratio (CBR) on the compactness, stability, and strength of subgrade fillers under different substitution rates were systematically evaluated. Furthermore, the study investigates the variation in the performance of CSRA, including crushing value, 7-day unconfined compressive strength, 90-day splitting tensile strength, compressive rebound modulus, and scouring resistance. The results indicate that (1) when recycled materials are used for roadbed filling, they can meet the specified compactness and CBR value requirements at a 100% substitution rate. The key is to control the impurity content and maximum particle size during the crushing process. (2) When recycled materials are used in a pavement structure layer, their performance is significantly affected by the material source and substitution rate. CSRA produced from demolished old pavements and bridges exhibits better performance. It is recommended that the substitution rate should not exceed 80% for subbase layers and 50% for base layers. This research provides recommended substitution rates and material-selection guidelines for the scientific utilization of C&DW recycled materials for specific engineering applications. The study has practical significance for improving C&DW resource utilization, reducing engineering costs, and lowering carbon emissions.

## 1. Introduction

With the continuous renewal of infrastructure, highway reconstruction and expansion projects have generated huge amounts of C&DW, and its utilization has become a critical issue for the sustainable development of the industry. Processing C&DW into recycled aggregates (RAs) for use in roadbed filling, pavement base layers, and recycled concrete has been extensively studied and implemented both domestically and internationally. For example, in Belgium, Denmark, Germany, and other countries, municipal incineration bottom ash (MIBA) has primarily been used in road construction. France, the United Kingdom, the United States, and other countries have also introduced corresponding policies to promote the application of MIBA as a subgrade material [1]. Research indicates that RA, particularly recycled concrete aggregate (RCA), has become a viable alternative to natural aggregate, with its replacement rate being a core parameter affecting the economic and environmental benefits of a project.

However, the recommended replacement rates for RAs vary significantly across different countries. Table 1 summarizes the recommended values for different application scenarios based on selected domestic and international studies and specifications, clearly illustrating this variation, which ranges from 30% to 100%.

Analysis shows that the differences in recommended replacement rates among countries primarily stem from the following factors: (1) variations in waste sources and composition (e.g., the ratio of concrete to brick–concrete mixtures); (2) differences in processing techniques and quality control standards; (3) disparities in material performance requirements across national technical specification systems (such as ASTM in the United States, EN in Europe, and JTG in China); and (4) variations in research priorities and engineering contexts. Currently, there is no international consensus on the optimal replacement rate for RAs, which hinders their large-scale and standardized application.

Domestically, Zhang [10] compared the performance of cement-stabilized mixtures containing natural aggregates and recycled construction waste aggregates. They also constructed a test section using a recycled aggregate inorganic mixture to determine the technical feasibility of applying recycled aggregate to graded gravel layers, subbases, and bases of municipal roads.

Fan [11] verified that pavement base layers constructed with recycled materials have sufficient strength, stiffness, and stability by designing lime fly ash-stabilized recycled materials and cement fly ash-stabilized recycled materials with different mix ratios.

Kuttah [12] assessed fourteen asphalt mixtures using the VTI’s circular road simulator test, which enables the evaluation of different road qualities, including surface deterioration and wear, friction, and visual inspection.

Mo [13] mixed recycled aggregates with natural aggregates and cement at different substitution rates to prepare CSRA base materials. The results showed increasing the recycled aggregate content reduces the mechanical properties of gravel materials. The compressive strength and elastic modulus of the recycled gravel materials decreased slowly at first and then rapidly as the recycled aggregate content increased.

Wu [14] used brick–concrete recycled aggregate to experimentally investigate a cement-stabilized base. The results showed that, compared with natural aggregates, the compressive strength and splitting strength of the mixture initially increased and then decreased with increasing recycled aggregate content.

Madina [15] investigated a cellulose-containing additive (CCA) derived from recycled waste paper as a renewable stabilizer for SMA, aiming to minimize binder drain-down while maintaining mechanical performance. The recycled paper-derived cellulose successfully replaced conventional stabilizers, offering comparable performance with a lower environmental impact and supporting circular-economy practices in pavement engineering.

These studies indicate that the strength, stiffness, and other properties of cement-stabilized recycled materials follow specific patterns with changes in RA content, and that excessive content leads to an attenuation in mechanical properties. Although these studies have confirmed the general technical feasibility of using recycled materials, most have focused on specific materials or localized performance. Consequently, there is a lack of quantitative research on the impact of substitution rate on road performance under the current Chinese standard system, including the “Technical Code for the Utilization of Building Solid Waste in Highway Engineering”, particularly for the complex solid waste sources used for expressway reconstruction and expansion projects. Consequently, the existing literature fails to provide universally applicable replacement rate recommendations.

In view of this, based on a highway reconstruction and expansion project in Guangdong Province, this study systematically analyzes the influence of RA substitution rates on the performance of subgrade filling (compaction characteristics and CBR strength) and CSRA (unconfined compressive strength, resilient modulus, and erosion resistance). Based on these analyses, we propose a recommended recycled aggregate replacement rate suitable for the reconstruction and expansion of expressways in China. This will provide a technical basis for balancing engineering costs, solid waste treatment costs and road performance requirements, thereby improving the economic and environmental sustainability of C&DW resource utilization.

## 2. Materials and Methods

### 2.1. Systematic Literature Review Method

To ensure the comprehensiveness and repeatability of data collection, firstly, combination keywords such as “recycled aggregate”, “construction and demolition waste”, “substitution rate”, “roadbed”, and “subgrade” were searched in the Web of Science, Scopus, and CNKI databases. The search covered recent research published from 2010 to 2026, with particular focus on articles published since 2020. The inclusion criteria comprised empirical studies, including experimental or engineering applications, in which quantitative data on recycled aggregate replacement rate and road performance were clearly reported. The data included key parameters such as literature source, waste type, aggregate treatment process, replacement rate, compaction degree, CBR value, unconfined compressive strength, and anti-erosion performance, thereby ensuring the reliability and consistency of the subsequent analysis.

### 2.2. Data Extraction and Classification Framework

First, according to the performance requirements, mechanisms, and application scenarios of C&DW recycled materials in different road structural layers, the data were divided into two categories: non-bonded recycled materials and CSRAs. Non-bonded recycled materials refer to RAs that are not stabilized by cement, lime, or other cementitious materials and are mainly used for subgrade filling. CSRAs refer to RAs mixed with cement and water for use in stabilizing pavement base or subbase layers.

Secondly, parameters reported in the literature were extracted. Basic information included the researchers, publication years, and sources. Material characteristics included waste sources, proportions of the main components, and aggregate treatment processes. Mixture design information includes RA replacement rate, cementitious material dosage, maximum particle size, and gradation. For the key performance test results, non-adhesive materials were evaluated primarily in terms of OMC, MDD and CBR, whereas cement-stabilized materials were evaluated using parameters including compression value, unconfined compressive strength, splitting strength, compressive rebound modulus, and erosion resistance.

Finally, the units of all physical quantities were standardized according to the International System of Units. The test standards on the performance data were based were marked, and outliers that deviated significantly from the overall trend without a reasonable explanation were identified and marked. Some representative data sources are shown in Table 2, where MRA denotes mixed RA, RCM denotes recycled clay masonry, RFA denotes recycled fine aggregate, and RBCA denotes RA from brick–concrete waste.

### 2.3. Brief Comparison of Domestic and International Relevant Specifications

The technical requirements for subgrade fill materials, such as CBR, maximum particle size, and degree of compaction, in the Chinese specification JTG D30-2015 [34] were generally consistent with those of international mainstream specifications like AASHTO and EN in terms of core performance indicators. All of these specifications aim to ensure the strength, stability, and durability of the subgrade.

However, there are certain differences in specific control parameters, which are mainly reflected in the limits for fine-grained soil content, permissible organic matter content, and additional requirements for special soils. While incorporating international experience, the Chinese specification places greater emphasis on adapting to the complex and varied geological conditions and regional material characteristics within China. Consequently, its technical clauses demonstrate a degree of flexibility and inclusiveness. This flexibility provides a potential technical window and adaptation space for the compliant application of non-traditional recycled fill materials, such as C&DW, in subgrade engineering. However, it also necessitates more detailed performance verification and design adjustments based on material characteristics in practical projects.

## 3. Results

### 3.1. Influence of Replacement Rate on the Performance of Subgrade Filler

#### 3.1.1. Technical Requirements of Subgrade Filler

The application of C&DW as a roadbed filler was one of the earliest areas of research in the field of C&DW utilization in road engineering. According to the requirements of JTG D30-2015, the technical index requirements should meet the requirements listed in Table 3.

The particle size of the filler has a significant influence on the compactness and stability of the subgrade. The larger the particle size of the filler, the greater the porosity, and the more filler is required, thereby increasing the construction cost and time. In addition, the larger the particle size of the filler, the greater the difficulty of compaction. Therefore, in the roadbed filling process, it is necessary to strictly control the particle size of the filler to ensure the compactness and stability of the roadbed. The CBR value is an important mechanical index of road subgrade materials obtained through testing, and plays a significant role in determining the quality of the subgrade. If the impurity rate is too high, this will affect the quality of roadbed filling. In addition to the data presented in the table, the specification also requires the roadbed filler to have good compaction performance. The maximum particle size and impurity rate can be controlled by crushing and screening processes, and the compaction performance and CBR value need to be measured according to the relevant test methods. A large number of studies have conducted experimental research on the feasibility of C&DW as a roadbed filler; therefore, this paper analyzes its road performance.

#### 3.1.2. Compaction Performance of Solid Waste Recycling

The compaction characteristics of C&DW roadbed fillers are usually characterized by MDD and OMC, and these values are obtained via a standard compaction test. MDD and OMC serve as important parameters for construction quality control for subgrade filling, aid in the establishment of compaction test standards for C&DW mixtures, and act as important parameters in standard compaction and bearing ratio tests.

**Figure 1:**
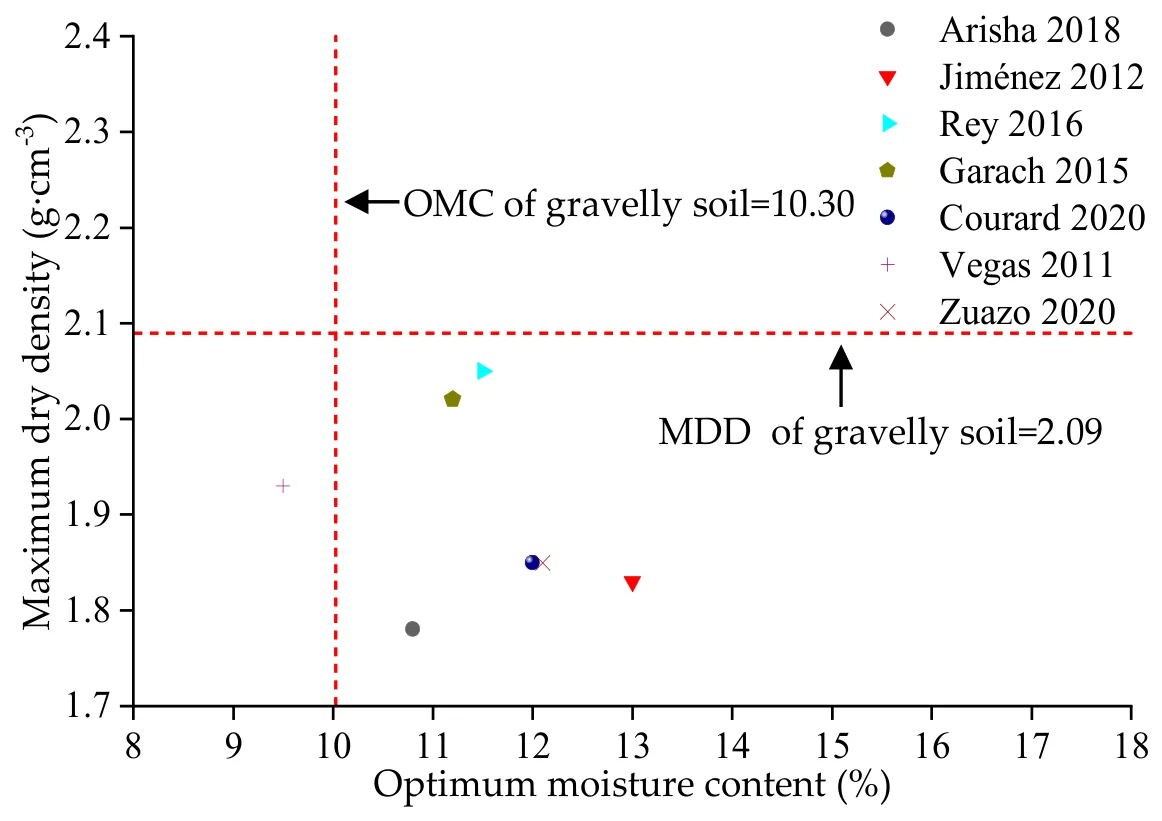
Statistics on MDD and OMC.

MDD and OMC are important parameters for subgrade construction. Scholars have extensively investigated C&DW recycled materials, and a summary of the test data reported by Arisha [16], Jiménez [17], Rey [18], Garach [19], Courard [20], Vegas [21], and Zuazo [22] is shown in Figure 1.

Article 3.3.3 of the JTG D30-2015 recommends the use of well-graded sandy gravel soil as an embankment fill material. However, “gravelly soil” itself is a broad category, and its OMC and MDD are influenced by various factors such as particle gradation, mineral composition, and plasticity index, resulting in significant numerical dispersion in practical engineering. Therefore, using it as a benchmark for recycled materials may lead to misleading evaluations of the performance of recycled fill materials.

Figure 1 shows that the compaction parameters of C&DW recycled fill materials are widely distributed (MDD: 1.78–2.21 g/cm^3^, OMC: 9.5–15.5%), directly reflecting the inherent heterogeneity caused by diverse raw material sources and complex compositions (varying proportions of bricks, concrete, and mortar). Compared with common natural sandy gravel soils, recycled fill materials exhibit a systematic trend of higher OMC and lower MDD. This indicates differences in compaction mechanisms, and suggests that their performance is primarily regulated by material composition—the higher the proportion of waste bricks, the greater the OMC and the lower the MDD. In engineering practice, rather than relying on an “idealized gravel soil” as a single benchmark, it is essential to conduct tests based on specific material sources and emphasize the variability and composition-dependent behavior of recycled materials.

**Figure 2:**
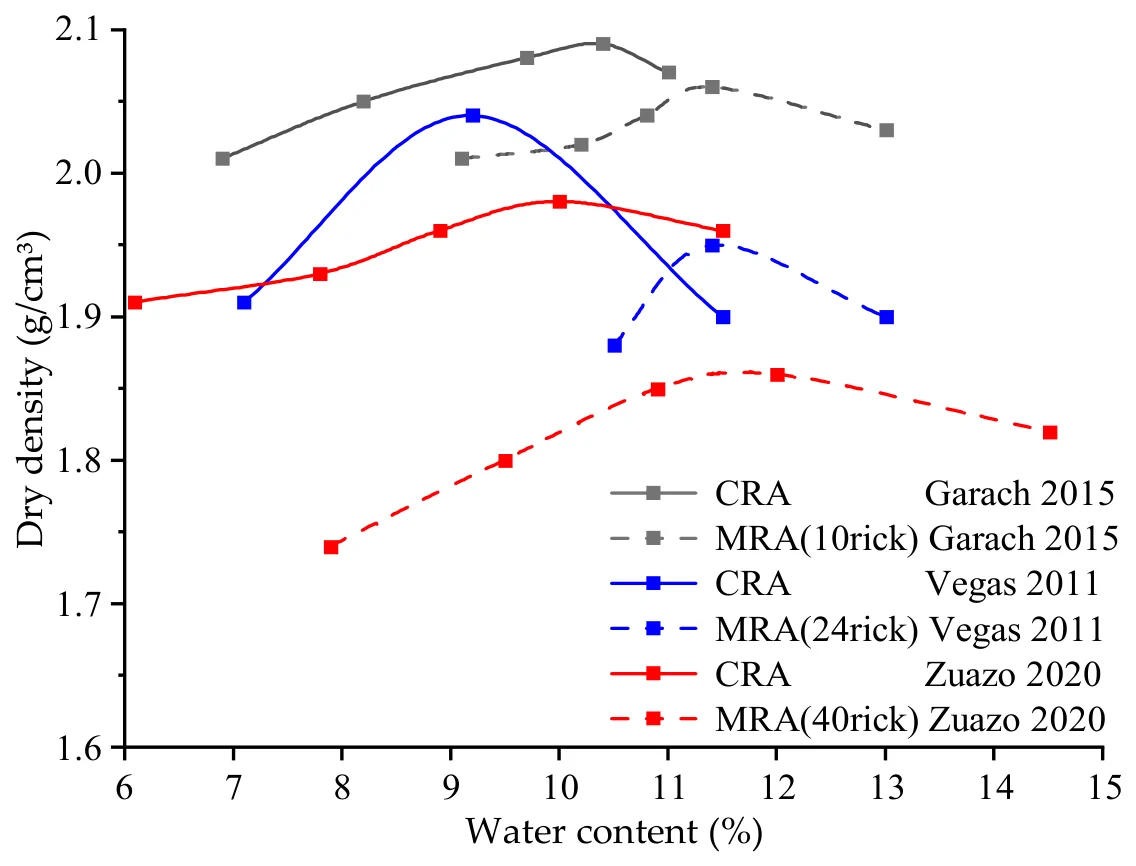
Compaction curves of C&DW with different compositions.

In order to explore the influence of brick content on the compaction characteristics of fillers, Garach [19], Vegas [21], Zuazo [22], and other scholars compared the compaction properties of fillers made of waste concrete aggregates (CRAs) and mixed C&DW aggregates (MRAs), respectively. The test results are shown in Figure 2.

From Figure 2, it can be seen that under the same experimental conditions, the subgrade filler made of CRA has a larger MDD and a smaller OMC than the subgrade filler made of MRA. The difference between the MDD and the OMC in the recycled aggregate is mainly due to the density and strong cement absorption of the material. Because MRA contains more waste bricks, and the brick surfaces have high porosity, resulting in strong cement absorption and low strength, the bricks are more likely to break under the action of external force, while the opposite is true for the subgrade filler made of CRA. When 10% waste brick was added, the MDD decreased by about 1.5%; when 20% waste brick was added, the MDD decreased by about 4%. When 40% waste brick was added, the MDD decreased by about 6%. It can be clearly seen that as the waste brick content increased, the MDD decreased gradually; therefore, waste brick content is an important variable affecting compaction performance.

**Figure 3:**
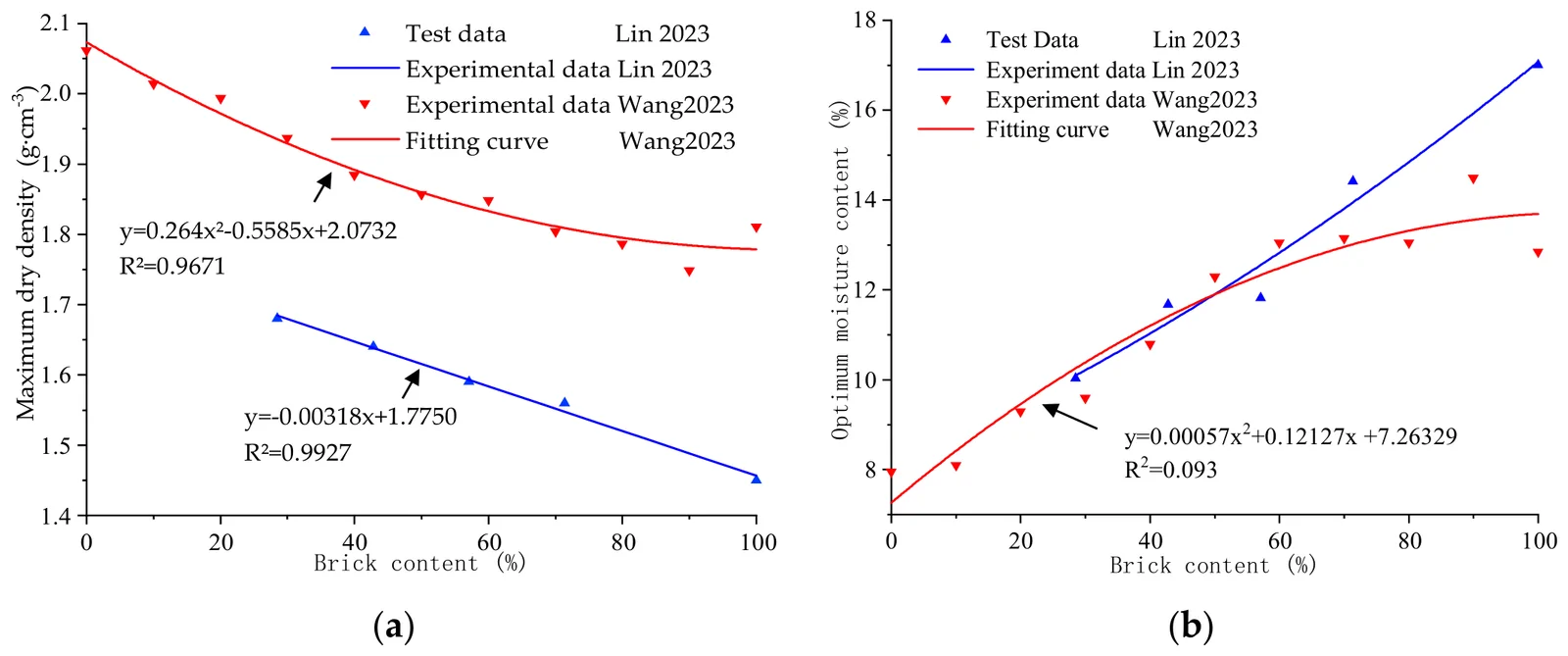
(**a**) Effect of brick content on MDD; (**b**) effect of brick content on OMC.

To further study the effect of waste brick content on the compaction performance of the filler, Lin [23] and Wang [24] established a simple prediction model for the recycled mixture through experiments. The test results are shown in Figure 3.

From Figure 3, it can be seen that there is a linear correlation between the compaction performance of C&DW fillers and the content of waste bricks. In general, the MDD of C&DW fillers decreases as waste brick content increases. From the fitting equations presented by various scholars, it can be seen that the MDD decreases by about 0.3 g cm^−3^ for every 10% increase in brick content. From the compaction results, it can be seen that when the waste brick content is 100%, the MDD is about 85% of that of C&DW fillers with the same waste brick content. When the proportion of waste bricks increases, the dry density of recycled fillers decreases, which is consistent with the test results of Garach [19] and Zuazo [22]. On the contrary, with an increase in brick content, the OMC of recycled filler increases continuously. When the brick content reaches 100%, the OMC increases by about 10%~70%. From the existing experimental data, the compaction performance of C&DW at different brick contents is good.

#### 3.1.3. Solid Waste Recycling Study of CBR

CBR is usually used to characterize the strength of subgrade filling. According to Table 1 of the JTG specification, a CBR value of greater than 8% indicates that the C&DW fill meets the regulatory requirements for subgrade filling in expressways. In practical engineering design, the target design value is typically set higher according to factors such as traffic load levels and subgrade conditions. The CBR = 8% criterion serves to establish a unified, standardized minimum performance benchmark. Its purpose is to provide a preliminary assessment of whether recycled fill meets the basic threshold for engineering application, rather than defining its optimal or final application scenario.

**Figure 4:**
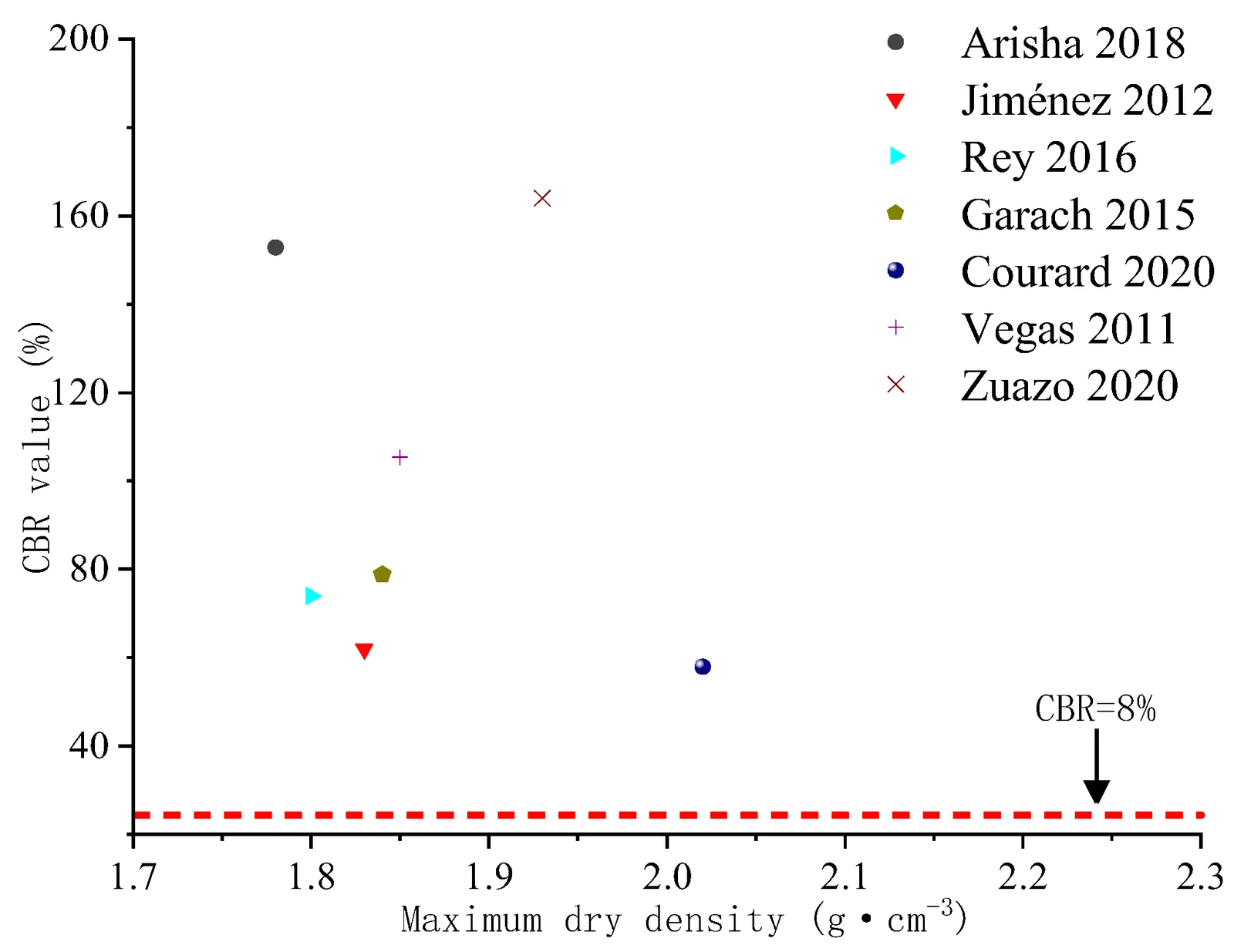
CBR values for C&DW.

**Figure 5:**
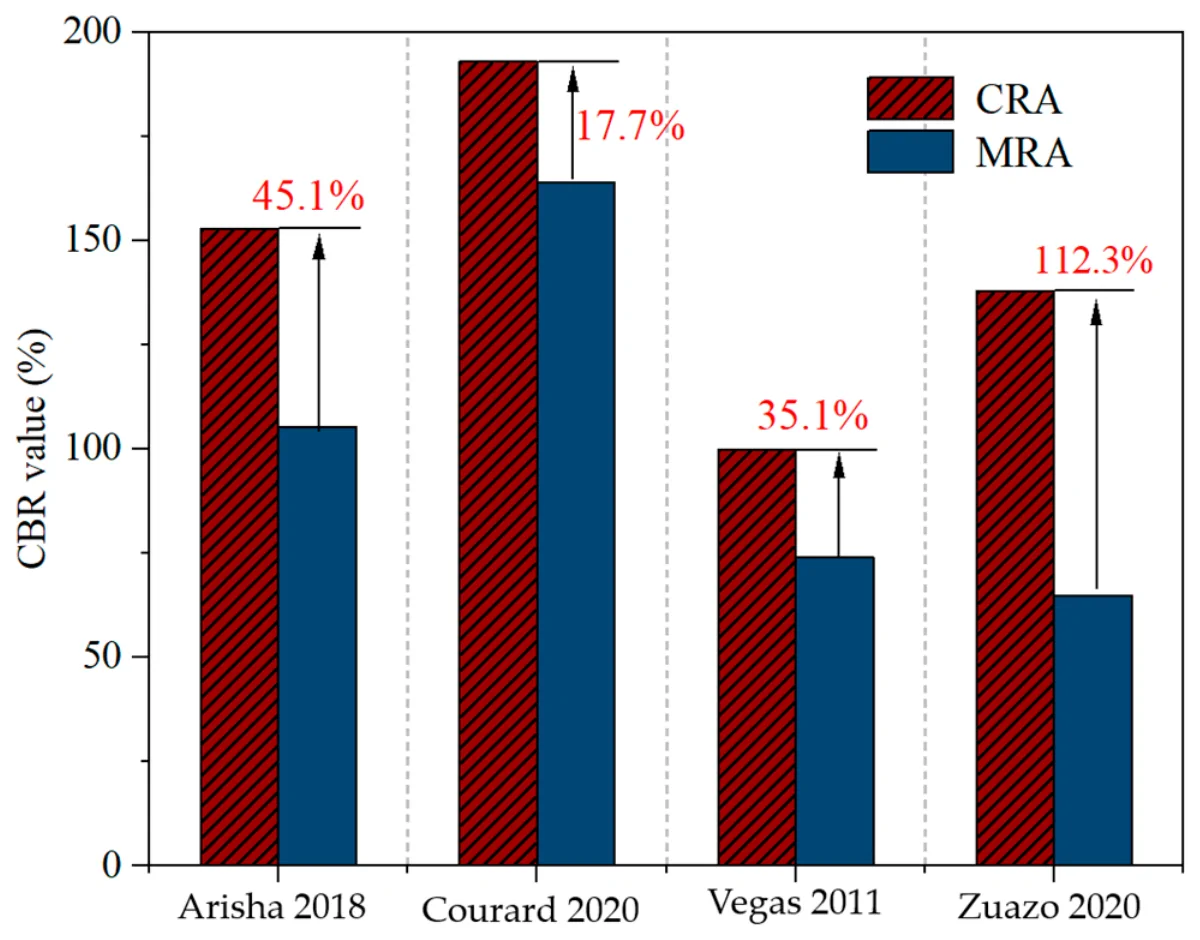
CBR values of fillers with different compositions.

A large number of scholars have tested the CBR value of C&DW fillers, and an analysis of the test data reported by Arisha [16], Jiménez [17], Rey [18], Garach [19], Courard [20], Vegas [21], and Zuazo [22] is shown in Figure 4. Several scholars have also compared the strength difference between the fillers prepared by CRA and MRA, as shown in Figure 5.

As shown in Figure 4, the CBR values of C&DW exhibit significant dispersion. When the filler consists entirely of waste bricks, the bearing capacity is the lowest, at only 34.7%; in contrast, when the filler is composed of CRA, the bearing capacity reaches its highest value of 195%. This result visually reflects the inherent extreme heterogeneity of C&DW materials. Therefore, meeting the minimum CBR requirement specified in the standards is merely a necessary condition for the material’s feasibility for application.

As shown in Figure 5, the CBR value of the CRA filler ranges between 100% and 197%, whereas that of the MRA filler is between 65% and 164%, indicating high variability. Furthermore, the CBR values of CRA fillers across different experiments show that they are 17% to 112.3% higher than those of MRA fillers, indicating significant dispersion. The average CBR value for CRA fillers is 162, compared with 104 for MRA fillers, demonstrating that for the same batch of construction solid waste, CRA fillers generally exhibit higher CBR values. However, horizontal comparisons reveal that the CBR value of some MRA fillers exceeds that of CRA fillers. For example, in the study by Courard [20], the MRA fillers achieved a higher CBR value than the CRA fillers in the other three experiments, indicating that optimizing the composition ratio of the mixed C&DW filler can achieve higher strength. This occurs because, although increasing the brick content is conducive to improving the compactness of backfill materials, the density of waste bricks is low, and an increase in compactness leads to a decrease in overall strength. Therefore, controlling the brick content is crucial for ensuring the quality of subgrade fillers.

**Figure 6:**
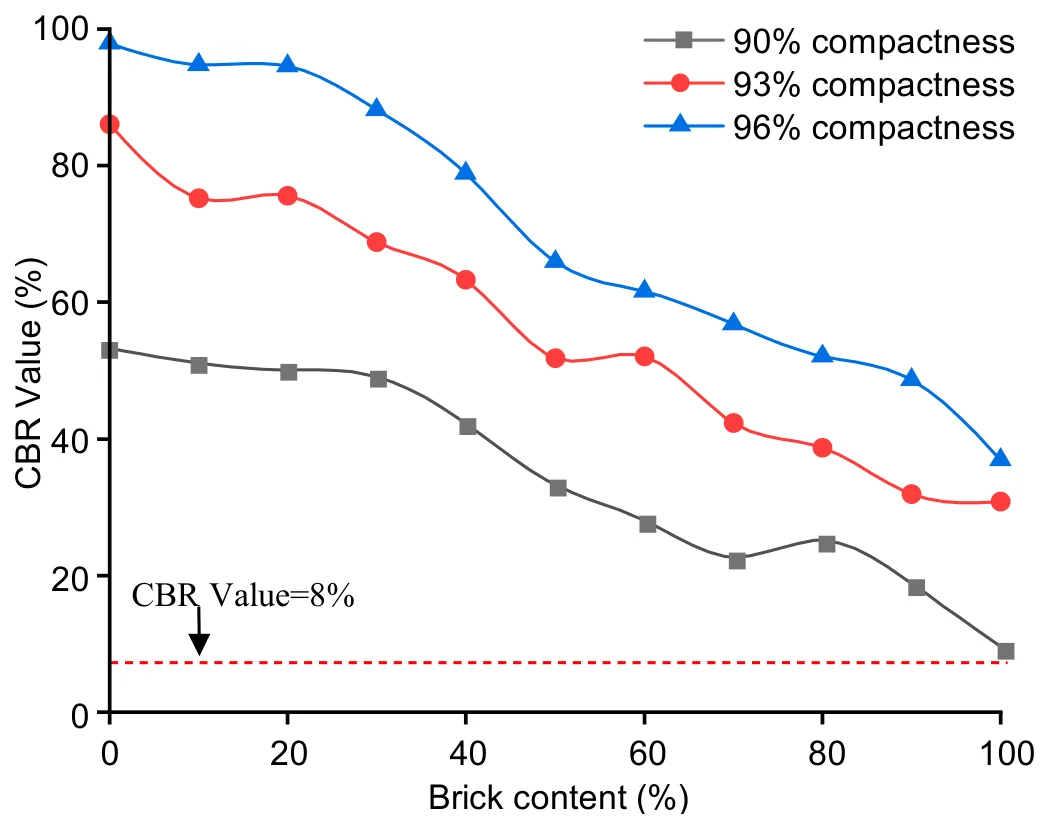
Effect of brick content on CBR values.

**Figure 7:**
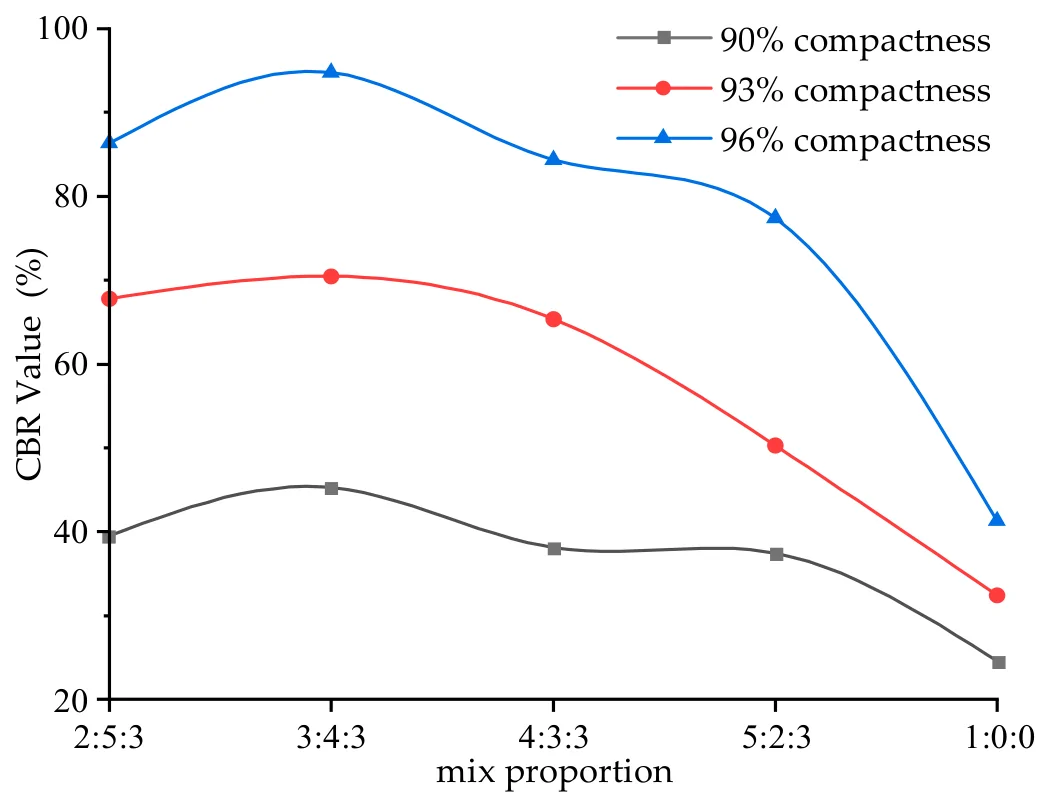
Effect of fit ratio on CBR values.

Lin [23] and Wang [24] and other researchers have investigated the influence of aggregate content on the strength of fillers, and a summary of their experimental data is shown in Figure 6 and Figure 7.

From Figure 6, it can be seen that the CBR value of the C&DW filler decreases with an increase in brick content under different compaction degrees. The CBR value of the C&DW filler with 100% brick content is about 80% lower than that of the C&DW filler with 100% concrete content. Although the performance significantly decreases, the required CBR value can still be met when the C&DW filler with 100% brick content is used for roadbed filling with strict quality control measures, such as limiting brick content and optimizing gradation. The main reason for the decrease is that the density and strength of bricks are lower than those of waste concrete. With an increase in brick content, the waste concrete content decreases, and the overall bearing capacity of fillers decreases, resulting in a low CBR value.

From Figure 7 and the test data, it can be seen that when the C&DW subgrade filler is mixed with mortar, the CBR value does not decrease with as brick content increases, as shown in Figure 6. When the brick/waste concrete/mortar ratio = 3:4:3, the mixtures with 90%, 93%, and 96% degrees of compaction exhibit their highest CBR values. The main reason for this is that due to the presence of mortar, when the brick content is relatively low, the mortar can fill the pores between large particles and improve the bearing capacity of the filler. When the brick content is higher, the content of waste concrete is lower, and the overall bearing capacity of the filler decreases, resulting in a decrease in the CBR value. The solid waste generated by the highway reconstruction and expansion project not only contains waste concrete and waste bricks, but also contains a large amount of mortar, meaning that the waste mortar generates more recycled powder in the aggregate production process. If it is applied to roadbed filling, it can greatly improve the utilization efficiency of C&DW.

Therefore, when designing C&DW for use as subgrade filler, the use of demolished old roads and bridges, or waste predominantly composed of discarded concrete, is recommended. Secondly, strict crushing, screening, and impurity control processes must be implemented during production to ensure the quality of the RA. If C&DW with a high brick–concrete content is used, or if quality control is inadequate, a 100% replacement rate may not meet engineering requirements, and the replacement rate must be reduced. It is recommended to prioritize the use of solid waste generated from building demolition, with a suggested bricks/waste concrete/mortar ratio controlled at 3:4:3.

### 3.2. Effect of Substitution Rate on CSRA Performance

#### 3.2.1. CSRA Technical Requirements

According to the requirements of JTG D50-2017 [36], the application of C&DW in expressway pavement base layers should meet the requirements listed in Table 4. Among these, erosion resistance is evaluated by a simulated rainfall–runoff erosion test (according to JTGE51-2009 [37]). A mass loss rate of less than 5% is identified as good, 5%~10% as medium, and more than 10% as poor. Due to the porosity of bricks, the erosion resistance of recycled fillers with low cement content generally needs to be improved.

The technical requirements for CSRA include crushing value, 7-day unconfined compressive strength (7d-UCS), 90-day splitting strength (90d-SS), compressive rebound modulus and erosion resistance. The crushing value is an important index for characterizing aggregate strength. The specification stipulates that aggregates used in cement-stabilized crushed stone mixtures should have a crushing value of no more than 26%. In order to ensure the usability and durability of the pavement structure, the semi-rigid base—as the main bearing layer of the pavement structure—must have sufficient strength. The 7d-UCS and the 90d-SS are strength performance indicators of the reaction material’s ability to resist vertical and horizontal loads. The specification also stipulates that under heavy traffic loads, the 7d-UCS of the pavement base and the subbase should reach 4 MPa and 2.5 MPa, respectively, and the 90d-SS should not be less than 0.4 MPa. The compressive resilient modulus describes the relationship between the stress and the rebound deformation of the material under a vertical load. The compressive modulus for the calculation of cement-stabilized macadam. Under the action of a vehicle, rainwater penetrates into the pavement structure through the cracks in the surface layer, and the base layer is scoured by the flowing cement. When the anti-erosion ability of the base layer is insufficient, the phenomenon of material loosening can occur, resulting in damage to the pavement structure. The existing specifications do not provide specific numerical requirements for anti-erosion performance, and CRSA is therefore required to demonstrate adequate anti-erosion performance.

**Figure 8:**
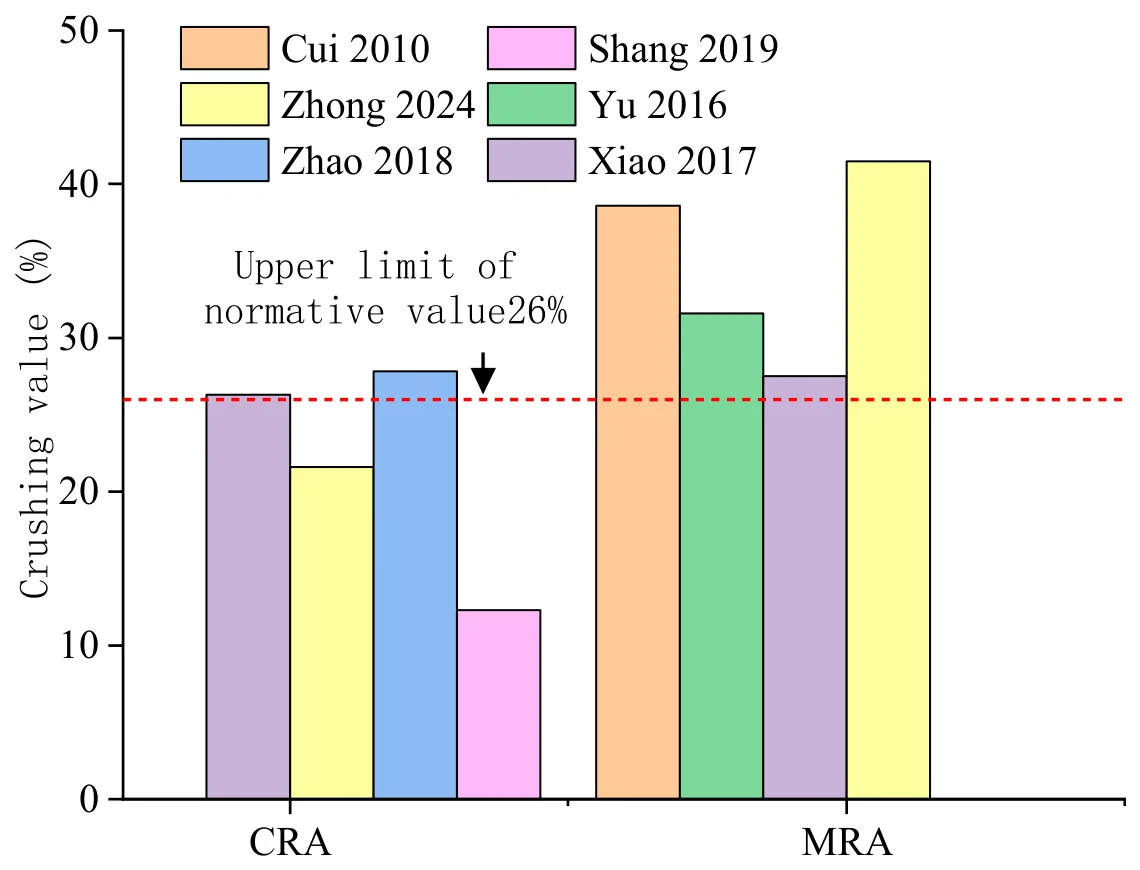
Statistics of crushing values of fillers with different compositions.

**Figure 9:**
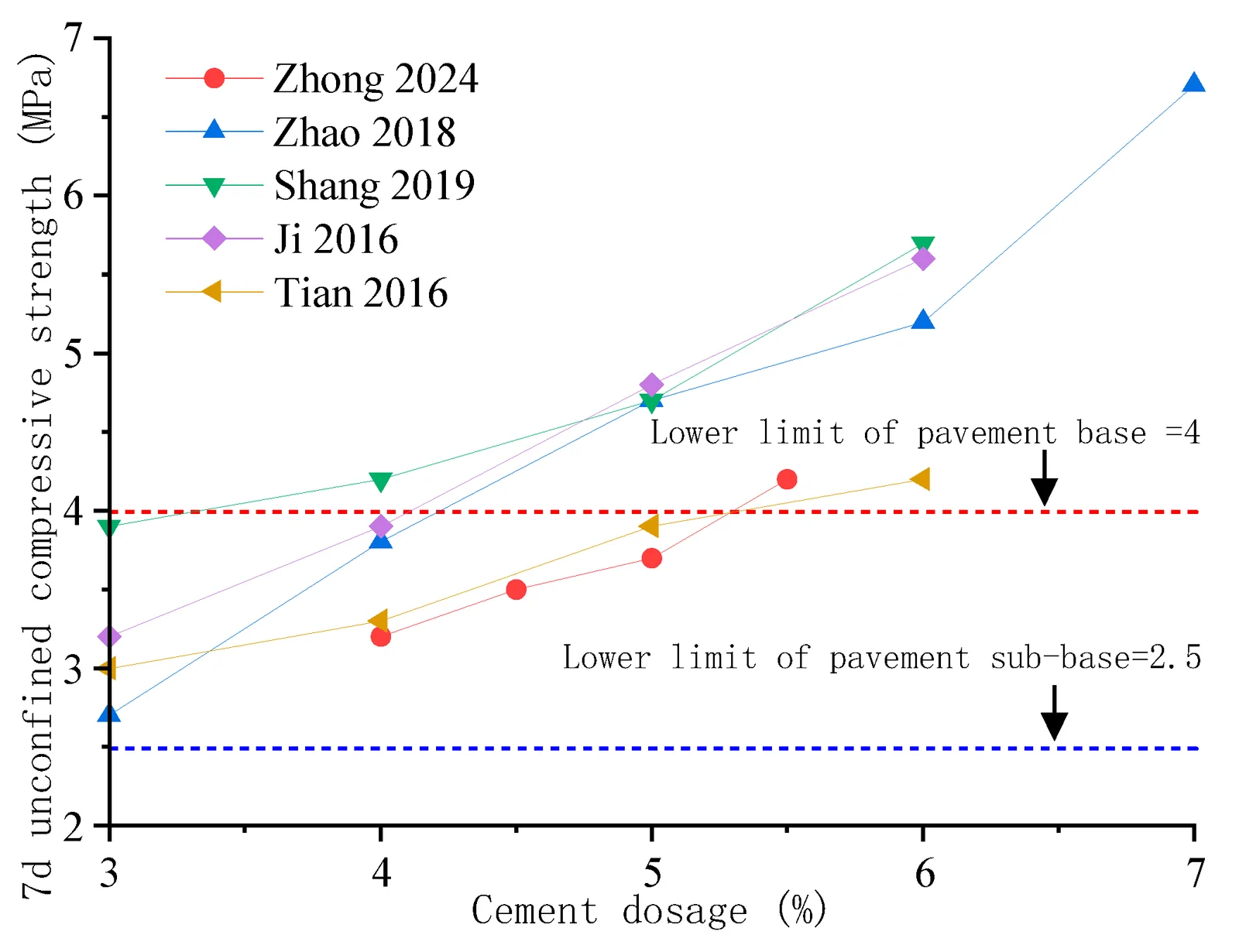
Effect of cement dosage on the strength of MRA.

Cui [25], Zhong [26], Zhao [27], Shang [28], Yu [29], Xiao [30], Ji [31], and Tian [32] studied the performance of recycled C&DW aggregates applied to pavement base layers. In order to facilitate an assessment of its applicability, the test data were summarized and compared, as shown in Figure 8 and Figure 9.

Figure 8 shows that the MRA crushing value is generally greater than 26%. This is because there are voids or microcracks within and on the surface of the brick slag, and their strength is low, making them easy to crush. Consequently, the crushing value, which ranges between 27.5% and 41.5%, is too large, with a high level of fluctuation. Due to the different years in which buildings were demolished and constructed in various regions, the degree of weathering of brick and tile materials also varies, and the aggregate strength produced after processing is affected accordingly. It is stipulated that the crushing value of stone used for highway bases should be less than or equal to 26%, and the crushing value of the brick aggregate clearly does not meet the requirements for pavement base and subbase materials in JTG D50-2017. CRA generally meets these requirements, with a crushing value ranging from 12.3 to 27.8%; the maximum value does not exceed the upper limit of the specification by 10%.

On the other hand, Figure 9 shows that the 7d-UCS of the MRA is greater than 2.5 MPa at 4% cement dosage, which meets the technical requirements of applying it to the subbase. However, to meet the 7d-UCS requirement for application to the base, a large amount of cement needs to be added for reinforcement, and this results in poor cost-effectiveness.

**Figure 10:**
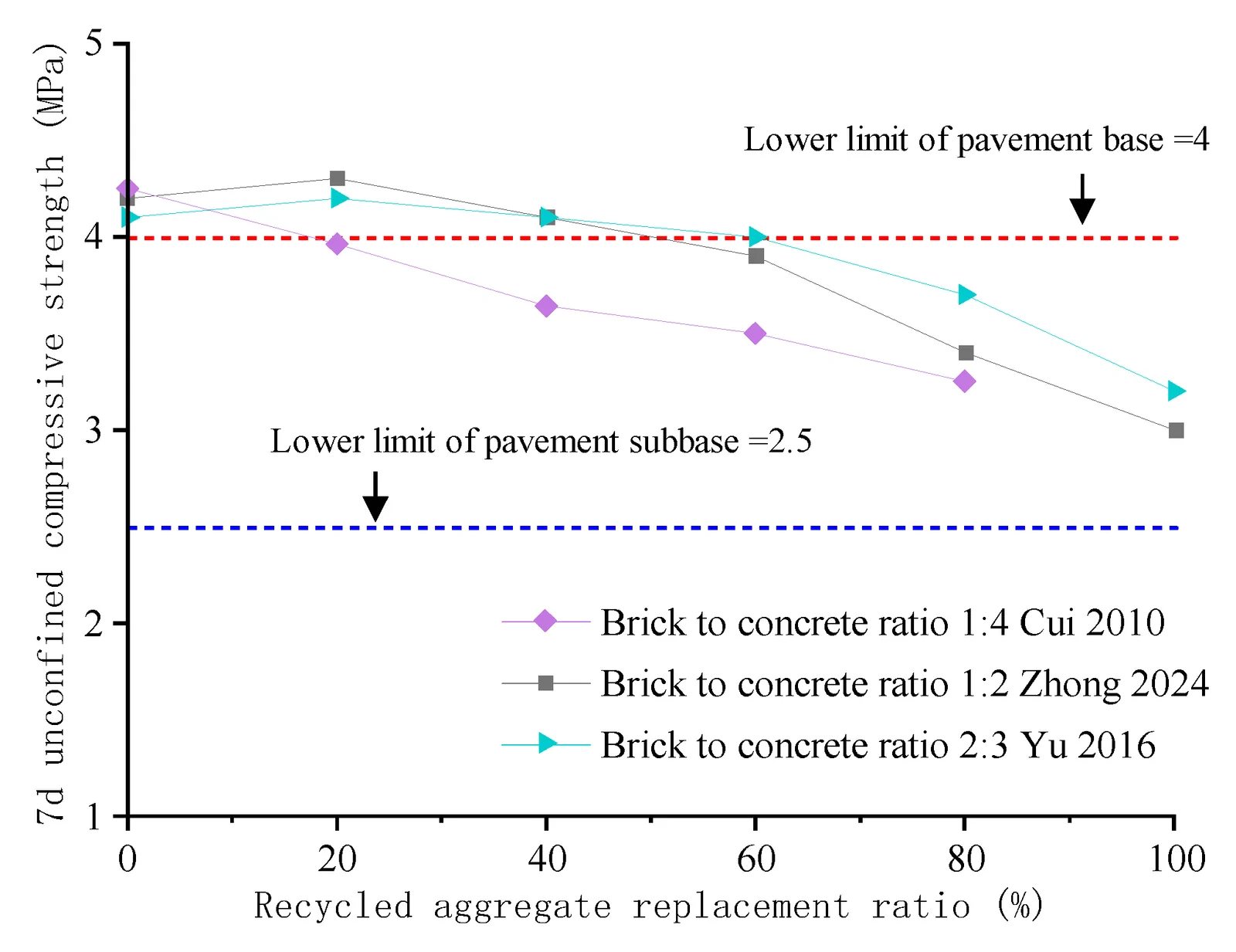
Effect of recycled aggregate dosage on compressive strength of MRA.

**Figure 11:**
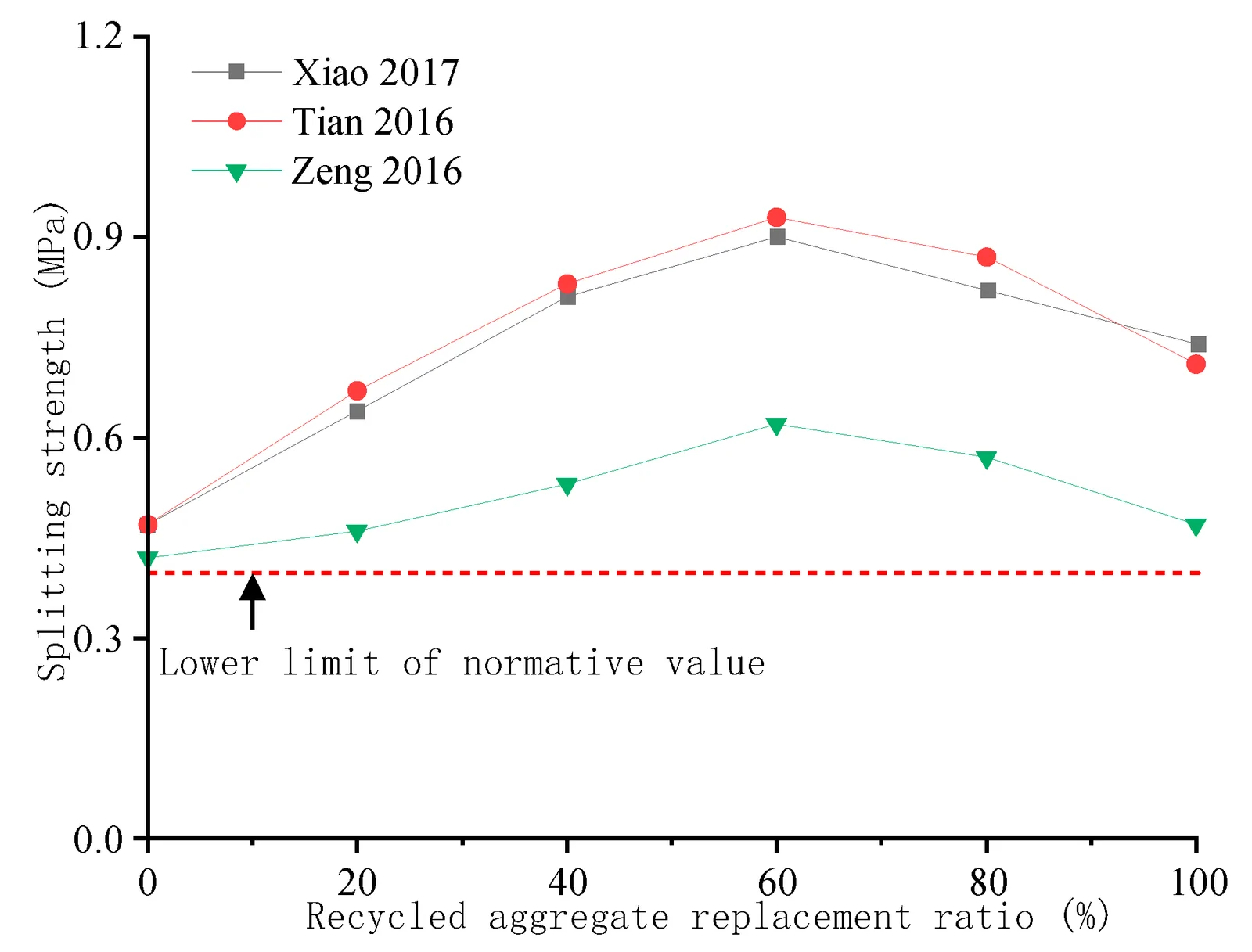
Effect of recycled aggregate dosage on splitting strength of MRA.

In the studies by Xiao [30], Tian [32], and Zeng [33], a cement dosage of 5% was used, and the influence of different solid waste aggregate mixing ratios on the strength performance of CSRA was studied. Their test data are summarized in Figure 10 and Figure 11.

Figure 10 shows that, on the one hand, from the perspective of the standard value requirements in the specifications, for MRA, when the replacement rate reaches 20%, the material fails to meet the standard requirements; therefore, MRA is not recommended for use in applications covered by these specifications. On the other hand, with respect to the standard value requirements for the subbase, when the brick–concrete ratio is low, the 7d-UCS of CSRA shows a trend of first increasing and then decreasing. This is because when the amount of recycled aggregate is small, although the overall strength of the aggregate decreases, its surface is rough, and the surface of the waste concrete is covered with mortar, containing some incompletely hydrated cement, which enhances its bonding strength. When the brick–concrete ratio is 1:0, the 7d-UCS of CSRA decreases rapidly with an increase in recycled aggregate content. When the recycled aggregate content increases from 20% to 60%, the 7d-UCS decreases by 43%. When the recycled aggregate content increases from 25% to 100%, the 7d-UCS decreases by 63%. By comparing the variation in strength under different brick–concrete ratios, it is found that as the brick–concrete ratio increase, its influence on 7d-UCS gradually increases. When the incorporation rate of recycled aggregate is 80%, the CSRA with brick–concrete ratios of 1:9, 2:8, and 3:7 decreases by 18%, 24%, and 29%, respectively, and all three meet the requirements of the specification for application in the subbase. However, in practical engineering, it is difficult to achieve accurate control of the composition ratio of MRA; therefore, it is not recommended to apply MRA to the subbase. Figure 11 shows that even in the most unfavorable case of a brick–concrete ratio of 1:0, when the replacement rate of the recycled aggregate reaches 100%, it can theoretically meet the requirements of the specification for splitting strength. The overall trend of splitting strength first increases and then decreases. The splitting strength is between 0.42 and 0.93, and the peak value occurs between 40% and 60% of the recycled aggregate content. Splitting strength indicates the ability to characterize the resistance to tensile failure; it is greatly affected by the bond strength of the cement mortar and has little to do with the strength of the aggregate itself. Therefore, an appropriate increase in the recycled aggregate can enhance the bonding force between the aggregate and the cement mortar, and thereby improve the splitting strength. Because the strength of the recycled aggregate is low and internal damage can easily occur during the crushing process, an excessively high incorporation ratio will lead to a decrease in its splitting strength.

**Figure 12:**
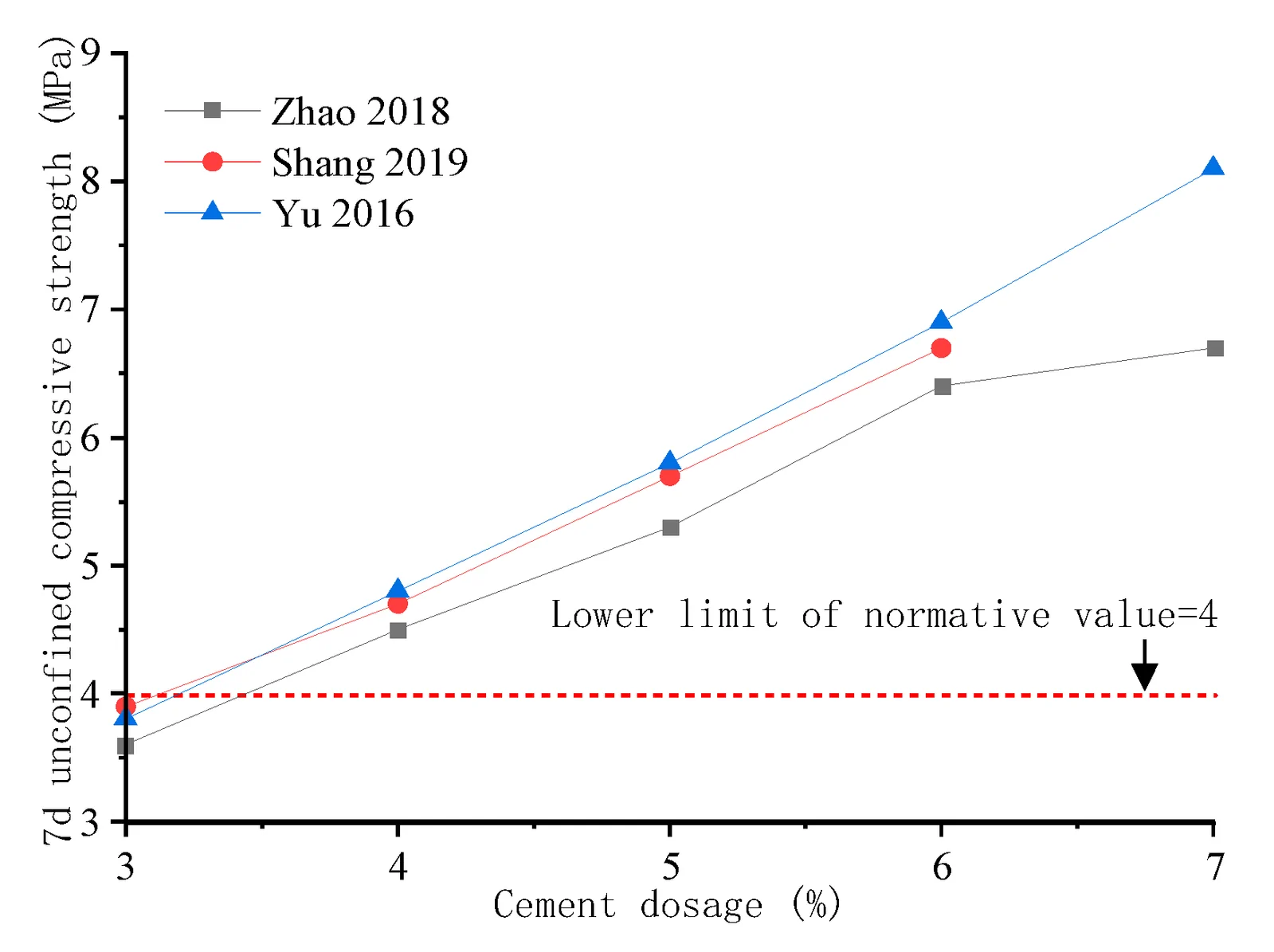
Effect of cement dosage on the strength of RAs.

**Figure 13:**
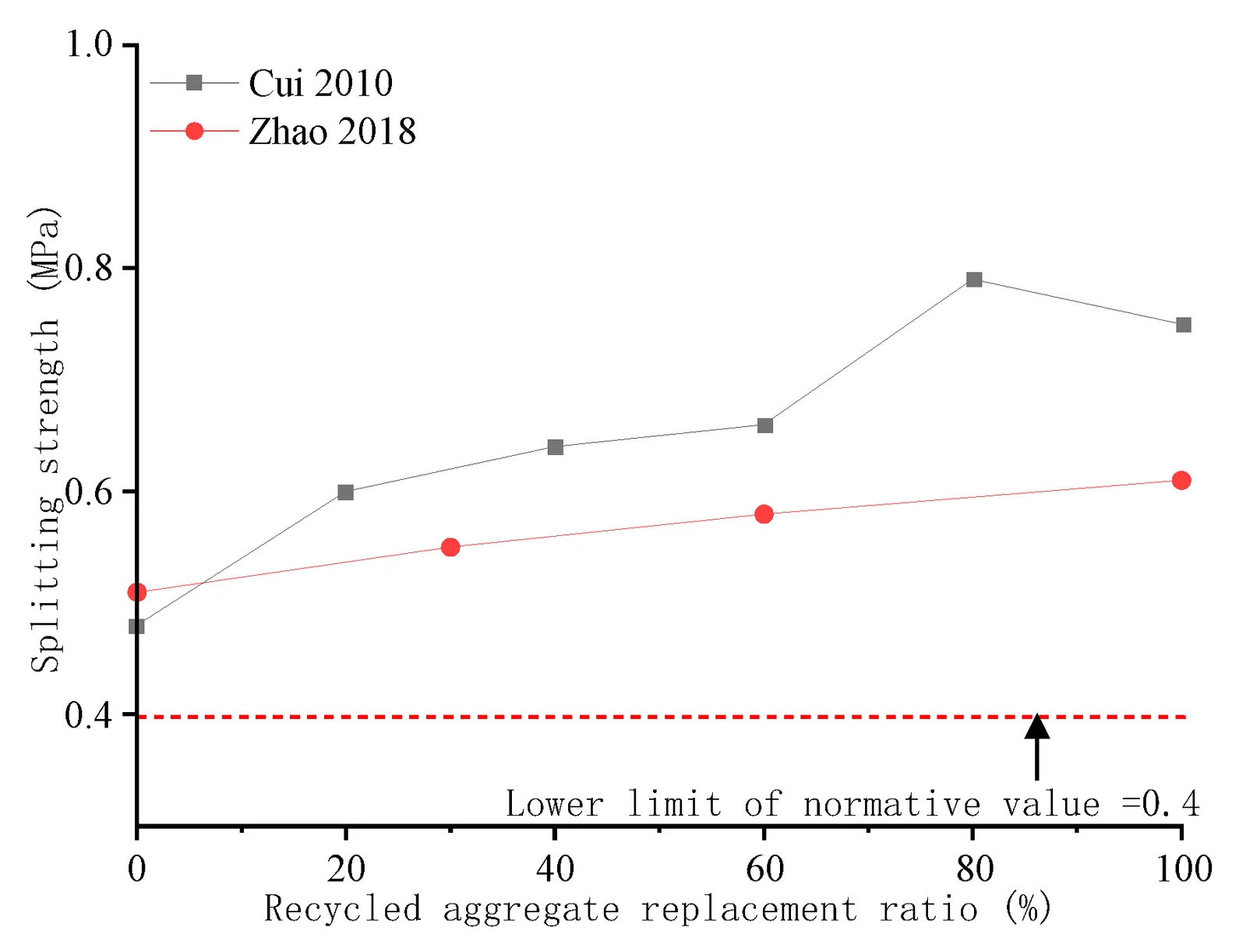
Effect of recycled aggregates dosage on splitting strength of RAs.

Zhao [27], Shang [28], and Yu [29] studied the strength performance of recycled aggregates produced by dismantling overpass components and waste cement concrete pavement panels for use in pavement base layers. Their test data are summarized in Figure 12 and Figure 13.

From Figure 12, it can be seen that at a 100% replacement rate of recycled aggregate, compared with the MRA cement-stabilized recycled aggregate, the recycled cement-stabilized aggregate prepared by such solid waste meets the 7d-UCS requirements outlined by the specification, while requiring a lower cement dosage. This is due to the high strength of the base materials in such solid waste, which results in higher-quality recycled aggregates, meaning that only a small amount of cement reinforcement is needed. On the whole, the change trend is the same as that of the MRA cement-stabilized recycled aggregate. With an increase in cement dosage, the 7d-UCS gradually increases, basically showing a linear growth. For every 1% increase in cement dosage, the 7d-UCS increases by about 1 MPa. Figure 13 demonstrates that the splitting strength gradually increases with an increase in recycled aggregate content, a similar trend to that of the MRA cement-stabilized recycled aggregate. When the recycled aggregate content is 100%, the splitting strength value increases by an average of 40%. Regardless of its content, the splitting strength values meet the specification requirements.

#### 3.2.2. CSRA Stiffness Performance

**Figure 14:**
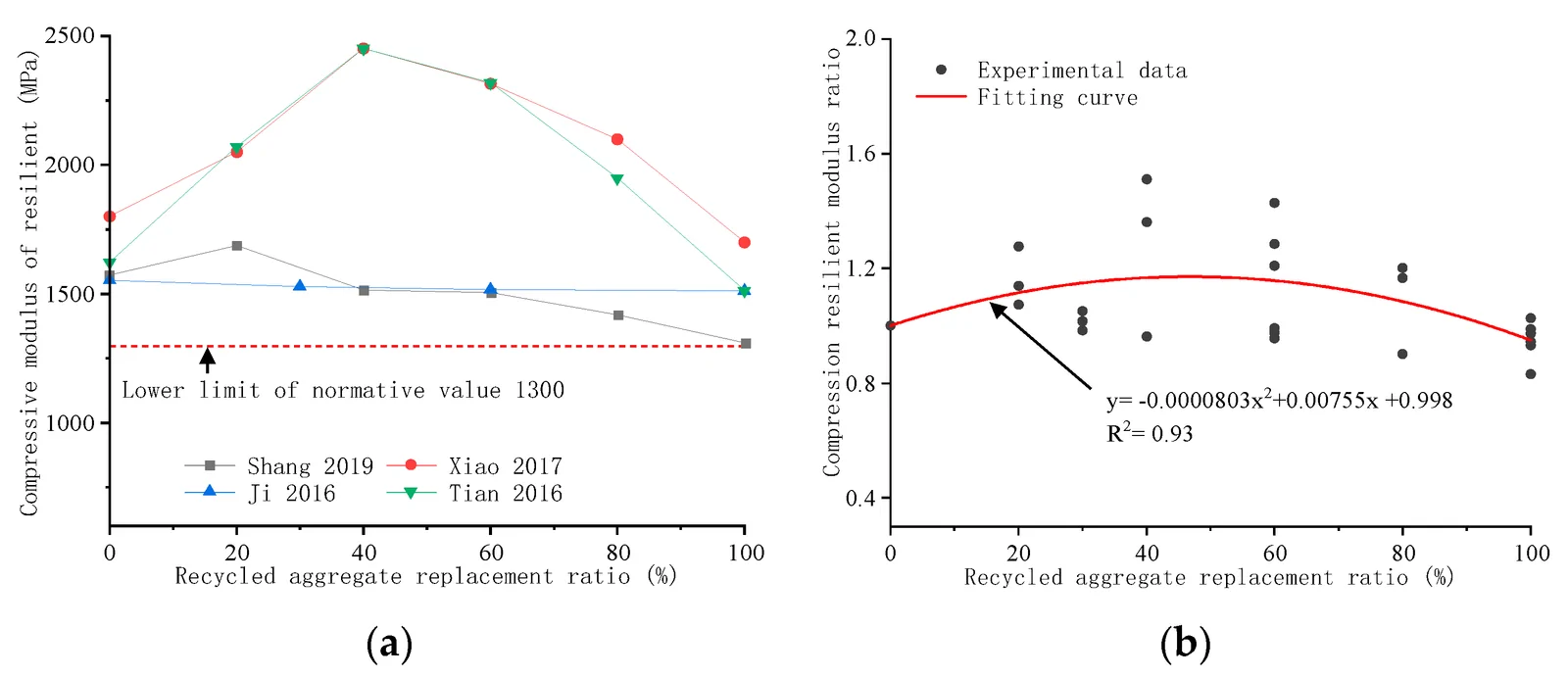
Effect of recycled aggregate substitution rate on compressive resilient modulus: (**a**) compressive modulus of resilience; (**b**) compressive resilient modulus ratio.

A summary of the test data of Shang [28], Xiao [30], Ji [31], Tian [32], and other scholars is provided in Figure 14a. In order to summarize the trends, the test data for all scholars except Hu Liqun were converted to the ratio of natural cement-stabilized aggregate in the control group, which is recorded as the ratio of compressive resilient modulus, as shown in Figure 14b.

From Figure 14, it can be seen that the influence of the replacement rate of the recycled aggregate on the compressive resilient modulus is closely related to the type of recycled aggregate. When CRA is used to replace the natural aggregate, the compressive resilient modulus first increases and then decreases with an increase in the replacement rate of recycled aggregate. This trend is due to the fact that, although the recycled aggregate derived from waste concrete is internally damaged due to crushing during the molding process, a greater amount of cement mortar remains attached to its surface, filling surface voids and increasing the bonding force. This improves the integrity of the CSRA and mitigates the adverse effects of the recycled aggregate. However, if too much recycled aggregate is added, the accumulation of these weak points will reduce the compressive rebound modulus.

In the analysis of the relationship between the recycled aggregate replacement rate and the compressive resilient modulus ratio, a goodness-of-fit R^2^ value of 0.93 was obtained. This indicates that the curve model has high statistical significance, and there is a significant linear negative correlation between the replacement rate and the modulus ratio, providing a quantitative basis for the rational selection of replacement rates in engineering applications.

#### 3.2.3. CSRA Anti-Scouring Performance

**Figure 15:**
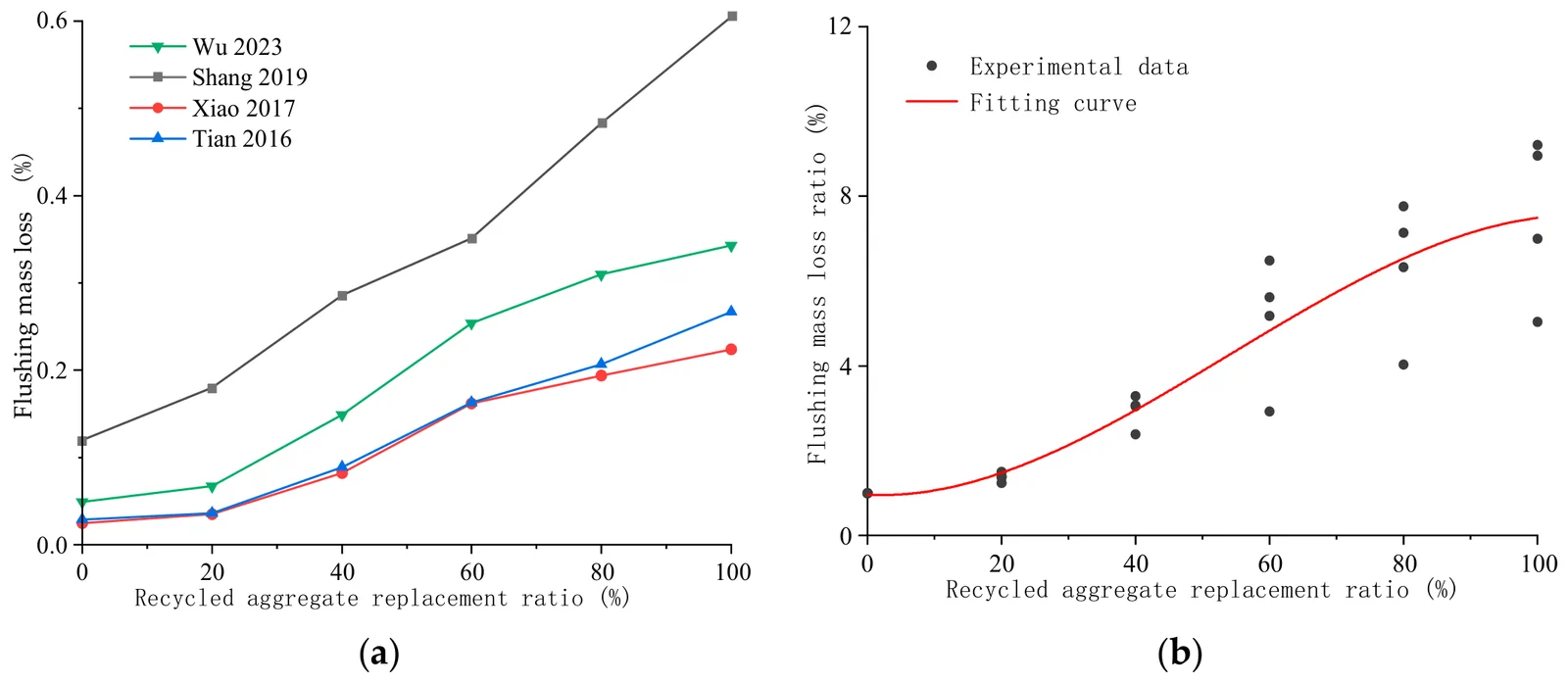
Effect of RA substitution rate on scour mass loss ratio: (**a**) flushing mass loss; (**b**) flushing mass loss ratio.

The experimental data reported by Wu [14], Shang [28], Xiao [30], and Tian [32] are summarized in Figure 15a. In order to identify the overall trend, the experimental data are converted into the ratio of the control group, which is recorded as the erosion mass loss ratio, as shown in Figure 15b.

As shown in Figure 15, as the RA replacement rate increases, the erosion mass loss of CSRA gradually increases. When the replacement ratio increases from 0% to 50%, the scour mass loss increases by approximately threefold, with the average scour mass loss ratio rising from 0.96% to 3.87%. When the replacement ratio further increases to 100%, the scour mass loss ratio reaches 7.49%, representing an increase of about 6.5-fold, which is less than the 10% mass loss rate specified in Table 4.

This is because a substantial amount of old cement mortar adheres to the surface of the RA, which results in a higher surface porosity. These pores adsorb more fine aggregates, which are easily washed away, resulting in an increase in erosion mass loss. This indicates that the RA replacement ratio significantly affects the scouring resistance of CSRA. Therefore, in practical applications, the RA content should be controlled to mitigate the adverse impact on scouring resistance. Furthermore, in base layers located in regions with high rainfall or frequent freeze–thaw cycles, scouring resistance is as important as strength. It is recommended that the replacement ratio for base layers be lower than that for subbase layers.

## 4. Discussions

### 4.1. Discussion of the Microscopic Mechanisms of Performance Variation

To further elucidate the evolution patterns of macroscopic properties (such as compressive strength and erosion resistance) resulting from changes in the recycled aggregate replacement ratio, this section analyzes the characteristics and mechanisms of the adhered mortar on the recycled aggregate surface and the resulting interfacial transition zone (ITZ) from a microscopic perspective.

Firstly, the old mortar adhered to the surface of recycled concrete aggregate (RCA) is characterized by high porosity and low strength, which is the fundamental cause of the deterioration in the material’s macroscopic performance [38]. Unlike natural aggregates, RCA introduces multiple ITZs within the cementitious matrix, including the interfaces between the original aggregate and old mortar and between the old and new mortar, with the latter being the weakest [39].

Secondly, as the RCA replacement ratio increases, the proportion of these weak ITZs within the system rises correspondingly. Scanning electron microscopy (SEM) observations indicate that material failure initiates at the ITZ, where cracks nucleate and subsequently propagate throughout the structure [40]. Nanoindentation tests further confirm that the elastic modulus and hardness within the ITZ region are significantly lower than those of the surrounding paste. This degradation in micromechanical properties accumulates with increasing replacement ratio, ultimately manifesting as a decline in compressive strength and erosion resistance [41].

When the replacement ratio exceeds 30%, the proportion of weak ITZs exhibits nonlinear growth, leading to an accelerated decline in macroscopic performance [40]. When the replacement ratio surpasses 50%, ITZ damage becomes the dominant failure mode, and simply increasing the cement content is insufficient to compensate for the performance loss caused by these microstructural defects [42].

In summary, the high porosity of the old mortar adhered to RCA and the multiple ITZ systems it introduces constitute the microscopic mechanism underlying the decline in macroscopic performance with an increasing replacement ratio. Controlling the replacement ratio or improving the ITZ structure through surface enhancement of the aggregates is crucial for ensuring material performance. It is recommended that the replacement ratio for base layers should not exceed 50%.

### 4.2. Research on Performance Assurance, On-Site Constructability, and Specification Compliance

The laboratory test results reported in the literature provide an important baseline for assessing the performance indicators of the potential application of RAs. However, when translating laboratory conclusions to large-scale field applications, key constructability issues must be considered.

There exists a non-negligible gap between laboratory density and field compaction. OMC and MDD determined in the laboratory are ideal values under specific compaction methods. In the field, factors such as the type of compaction equipment and lift thickness may limit the achievable degree of compaction, which could be lower than the laboratory value. For RAs with high water absorption, such as brick–concrete mixtures, moisture evaporation during construction may cause the actual moisture content to deviate from the OMC, thereby affecting compaction effectiveness. Therefore, rigorous verification using a field trial section is essential during construction.

Gradation defects can significantly impact workability. RAs often exhibit high water absorption and porosity, and they may suffer from insufficient fine particles or missing intermediate particle sizes due to the crushing process. Such poorly graded materials may achieve satisfactory density and CBR values in laboratory molds under standard compaction. However, during field spreading and rolling, they may appear loose, be difficult to shape, and be prone to excessive particle movement under compaction, failing to form a dense and stable structural layer. This explains why materials meeting laboratory specifications may exhibit poor workability on-site.

Index-type tests, such as CBR and unconfined compressive strength, are effective tools for material classification and preliminary design, but they cannot fully simulate the long-term performance of materials under complex conditions such as dynamic traffic loads and moisture variations. For instance, a poorly graded material that merely meets the CBR ≥ 8% requirement may still exhibit insufficient erosion resistance and fatigue performance. Therefore, compliance with specification indicators is a necessary condition for material application, not a sufficient one. For recycled materials, after passing conventional indicator screening, it is essential to conduct specialized tests that simulate actual service conditions, such as dynamic triaxial tests to determine resilient modulus and permanent deformation characteristics or fatigue tests to systematically verify their long-term durability, thereby compensating for the shortcomings of traditional empirical specifications.

In summary, conclusions regarding replacement ratios derived from literature data should be regarded as an assessment of performance potential under controlled laboratory conditions. In practical engineering, to ensure successful application, it is necessary to supplement material sourcing and mix design with specialized evaluations, gradation optimization, and long-term road performance assessments.

### 4.3. Limitations of the Study

This study primarily investigates the relationship between the road performance of RAs and their replacement ratio through a systematic literature review and secondary data analysis, which inherently entails certain limitations.

First, there is heterogeneity in the data sources. The integrated literature data originate from different countries, research teams, testing standards, and material sources, leading to variations in testing methods, performance indicators, and material descriptions. This inherent heterogeneity may introduce certain biases, potentially affecting the generalizability of the conclusions.

Second, there is a lack of original experimental validation. Independently designed experiments were not conducted to directly verify and deepen the understanding of key patterns. Consequently, the proposed recommended replacement ratios still require validation and calibration through field tests within specific engineering contexts.

Furthermore, the analysis of microscopic mechanisms is indirect. The discussion of the microscopic mechanisms underlying performance evolution primarily relies on observations from other studies, such as electron microscopy and nanoindentation results, constituting indirect evidence. Future research should employ direct microstructural characterization experiments to establish quantitative models linking microstructure with macroscopic performance for specific material streams.

Future work should focus on conducting targeted experimental studies, accumulating long-term field performance data, and developing performance-based design specifications and evaluation systems tailored to recycled materials.

## 5. Conclusions

Based on the context of China’s highway reconstruction and expansion projects and the JTG specification framework, and through a systematic literature review and data analysis, the performance and replacement rate optimization of C&DW recycled aggregates in subgrade filling and cement-stabilized base layers were investigated. The main conclusions are as follows:

- When recycled materials are used for subgrade filling, C&DW recycled aggregates can fully replace natural fill materials for highway subgrade construction under strict control of impurity content (≤1%) and maximum particle size (≤100 mm). A replacement rate of 100% is recommended.
- Material composition has a significant impact on compaction performance and CBR values. It is recommended to prioritize the use of concrete-type solid waste generated from the demolition of old roads and bridges. If demolition waste from buildings is used, the ratio of bricks, waste concrete, and mortar should be controlled at 3:4:3.
- For recycled aggregates used in subbase layers, the replacement rate should not exceed 80%; for base layers, it should not exceed 50%. Exceeding these thresholds requires compensation through mix-proportion optimization or material enhancement.
- When applying recycled materials to CSRA mixtures, the source of the recycled aggregates plays a decisive role in their performance. Recycled concrete aggregates (CRAs) from old roads and demolished bridges exhibit significantly better strength and durability than mixed demolition waste (MRA) containing bricks and mortar. The former is recommended for priority use.
- As the replacement rate increases, the erosion resistance of CSRA mixtures shows an exponential decline. In regions with heavy rainfall or frequent freeze–thaw cycles, the replacement rate should be strictly controlled, and the addition of anti-erosion modifiers should be considered.
- This study is mainly based on historical literature data. In the future, it is necessary to verify the performance of recycled materials under actual traffic and environmental loads through full-scale tests and long-term monitoring.

## Figures and Tables

**Table 1:** Summary of recommended substitution rates for recycled materials.

Serial	Country	Pavement Layer	Recycled Material Type	RSR
1	Japan [2]	Subgrade	RCA	100%
2	France [3]	Subgrade	Dredged sediments and RCA	Sediments ≤ 20%
3	India [4]	Subgrade	Red mud–fly ash	10–30%
4	India [5]	Granular subbase (GSB)	RA	≤60%
5	Spain [6]	Road base and intermediate layers	RCA/RMA	RCA ≤ 75%/RMA ≤ 35%
6	Turkey [7]	Road base	RCA	50–75%
7	Turkey [8]	Sustainable road base	RCA + BA	35%
8	China [9]	Sustainable road base	RA	≤30%

**Table 2:** Summary of literature data sources and key parameters.

Serial	Researcher	Waste Source	Application Scenario
1	Arisha [16]	RCA and RCM from C&DW	Pavement structure (UGMs)
2	Jiménez [17]	MRA from non-selected C&DW	Unpaved rural roads
3	Rey [18]	Unbound MRA from C&DW	Low-traffic unpaved rural roads
4	Garach [19]	RCA and MRA	Unbound road base and subbase
5	Courard [20]	RFA from C&DW	Unbound road subbase
6	Vegas [21]	MRA (concrete and ceramics)	Unpaved road structural layers
7	Zuazo [22]	RCA from C&DW	Sustainable road base
8	Lin [23]	RBCA	Roadbed filler
9	Wang [24]	RBCA	Subgrade backfill of metro vehicle section
10	Cui [25]	RBCA	Road base/subbase
11	Zhong [26]	RBCA	Expressway subbase
12	Zhao [27]	RBCA	Road base/subbase
13	Shang [28]	RBCA	Road base
14	Yu [29]	Waste cement concrete	Road base
15	Xiao [30]	RBCA	Road base
16	Ji [31]	RCA	Expressway pavement base
17	Tian [32]	RCA	Pavement base
18	Zeng [33]	RCA	Pavement base

**Table 3:** Technical requirements for C&DW roadbed fills.

Serial	Project	Project Classification	Unit	Specified Value	Specification
1	Maximum particle size	Roadbed	mm	≤100	JTGT 2321-2021 [35]
		Embankment	mm	≤150	
2	Impurity rate	Subgrade	%	≤1	JTGT 2321-2021
3	CBR value	Roadbed (0~0.3 m)	%	8	JTG D30-2015
		Roadbed (0.3~0.8 m)	%	5	
		Embankment (0.8~1.5 m)	%	4	
		Embankment (>1.5 m)	%	3	

**Table 4:** Technical requirements for the application of C&DW to CSRA.

Serial	Project	Unit	Structural Layer	Prescribed
1	Crushing value	%	-	≤26
2	7-day unconfined compressive strength	MPa	Pavement base	4.0~6.0
		MPa	Pavement subbase	2.5~4.5
3	90-day splitting strength	MPa	-	≥0.4
4	Compressive rebound modulus	MPa	-	1300~1700
5	Erosion resistance	-	-	Adequacy

## Data Availability

The original contributions presented in this study are included in the article. Further inquiries can be directed to the corresponding author.
